# Development of a Novel Deep Learning-Based Gaze Estimation Method for Detecting Strabismus

**DOI:** 10.7759/cureus.104035

**Published:** 2026-02-21

**Authors:** Midori Watabe, Hiroki Nishimura, Rohan J Khemlani, Shinri Sato, Shintaro Nakayama, Eisuke Shimizu

**Affiliations:** 1 Chemical Engineering, United World Colleges International School of Asia, Karuizawa, Karuizawa, JPN; 2 Ophthalmology, OUI Inc., Tokyo, JPN; 3 Ophthalmology, Yokohama Keiai Eye Clinic, Yokohama, JPN; 4 Ophthalmology, Keio University School of Medicine, Tokyo, JPN

**Keywords:** artificial intelligence in ophthalmology, binocular vision, deep learning, gaze estimation, strabismus

## Abstract

Background

This study reports the development and preliminary validation of a deep learning (DL)-based algorithm capable of quantitatively estimating ocular alignment, specifically the direction and angle of eye position, using a technique known as gaze estimation. The purpose is to evaluate this algorithm as a novel method for detecting and quantifying strabismus.

Methods

A gaze-estimation model based on DL was applied to video input of ocular positions. The model is trained on a set of computer-generated eye images synthesized using UnityEyes (Computer Vision Laboratory, ETH Zurich, Zurich, Switzerland). The algorithm outputs visualizations of the right and left eyes along with estimated gaze angles. Two cases were examined: one without prior ophthalmologic history and one with a known diagnosis of exotropia. Additionally, the algorithm was applied to 10 subjects without ophthalmologic abnormalities to assess the correlation between the gaze directions of the left and right eyes.

Results

A total of 12 subjects were included in the study: two case subjects and 10 control subjects without ophthalmologic abnormalities. In Case 1 (no ophthalmologic history), the estimated gaze deviation was 4.3 degrees in the right eye and -0.5 degrees in the left. In Case 2 (diagnosed exotropia), the estimated deviation was 0.7 degrees in the right and -10.1 degrees in the left, closely reflecting the clinical diagnosis. Among the 10 control subjects, a strong correlation was observed between the gaze angles of both eyes (Spearman's r=0.961-0.965).

Conclusion

The algorithm demonstrated potential for quantifying strabismus angles through video-based gaze estimation. This method may offer a practical, non-invasive, and accessible approach for strabismus assessment, pending further validation against established clinical standards. Future work will enhance accuracy by incorporating multi-device datasets.

## Introduction

Strabismus (diplopia) is defined as a condition in which the right eye and left eye look at different places [1]. When strabismus occurs during infancy, when sensitivity to visual stimuli is high, the strabismic eye may be suppressed, impairing visual development and often resulting in amblyopia. This underscores the significant impact of strabismus on the development of visual function in children [2].

The recent proliferation of digital devices has raised concerns about the impact of so-called “smartphone strabismus” on children's eyes, and it has been reported that excessive use of digital devices is associated with myopia progression and inhibits proper development of binocular visual function [3]. A 2016 South Korea study reported the development of acute esotropia due to excessive smartphone use, and many similar reports have been observed in Japan [3,4].

Binocular vision abnormalities can be screened quantitatively to some extent [1] and are often detected in children during school vision screenings. In particular, with the widespread use of screening devices such as the Spot Vision Screener (SVS), there is an increasing number of cases referred to ophthalmologists when abnormalities are detected during pediatric examinations, beyond regular medical examinations [5]. However, current screening devices remain costly and not scalable in low-resource settings, motivating an AI-based video solution, such as SVS or proficiency in testing to quantify strabismus.

Recent technological advances have raised expectations for the application of artificial intelligence (AI) to the medical field. In ophthalmology, research and development of automated diagnosis and diagnostic support systems using AI has been active, especially in the diagnosis of fundus diseases using fundus photographs or other means [6]. However, AI-assisted detection and screening of strabismus remain in the research stage, facing several technical and practical challenges [6].

In this study, we report the development and preliminary validation of a deep learning (DL)-based algorithm capable of quantitatively estimating ocular alignment, specifically the “direction” and “angle” of eye position, using a technique called “gaze estimation” based on Stacked Hourglass Networks [7].

The objective of this study was to evaluate the feasibility of a novel method for detecting and quantitatively assessing strabismus by estimating ocular alignment, specifically the direction and angle of eye position, using DL-based gaze estimation. The proposed method applies a DL gaze-estimation model trained on synthetic eye images to video-derived ocular position images, enabling recognition of the right and left eyes and estimation of gaze angles in degrees based on iris landmark detection.

## Materials and methods

This study was conducted with the approval of the Kyoto Expert Ethics Review Committee (No. KEEC-21015).

**Figure 1:**
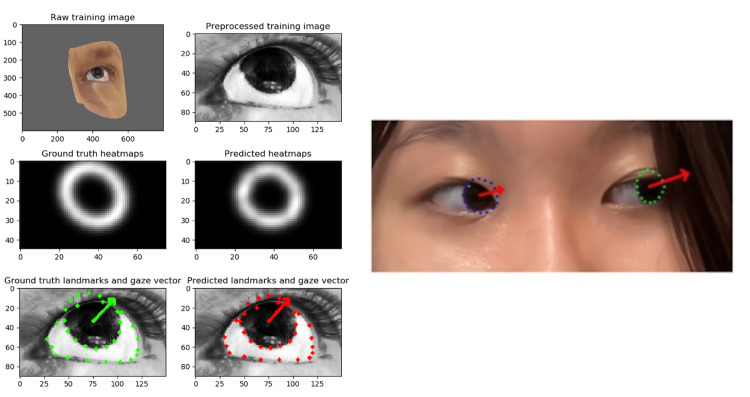
Gaze estimation model and example output with corneal region markings. (Left) Schematic diagram of the gaze estimation model. (Right) Output image (second author’s portrait) when an ocular position image is input. The right corneal region is enclosed by a blue dotted line, and the left corneal region by a green dotted line. The estimated gaze angle is indicated by a red arrow. Written informed consent was obtained for the publication of the participant’s images.

This developed algorithm uses a DL-based gaze estimation technique to quantitatively estimate ocular position. Utilizing the publicly available gaze estimation code (code for the repository of gaze estimation/pre-trained model) and the pre-trained model file available on GitHub [8], the program can recognize the features of the left and right eyes when an image is input. Specifically, it maps the shape of the irises in both eyes. Then, it calculates the direction of gaze in pitch and yaw format and outputs it as an angle in degrees (Figure 1). Visual Studio Code (Microsoft Corp., Redmond, WA, USA) was used as the code editor. When this program runs, it displays the irises with blue and green landmarks and overlays red arrows according to the estimated gaze angle (Figure 1).

The program was installed on a MacBook Pro 14-inch 2021 (MacBook; Apple Inc., California, USA), and the verification was conducted using the built-in camera. The program was activated using “Terminal,” a UNIX terminal emulator that comes standard with Apple's macOS, and the output image was set to display blue (right eye) and green (left eye) landmarks according to the subject's iris position and red arrows according to the estimated gaze degree on the screen obtained with the built-in camera (Figure 1).

**Figure 2:**
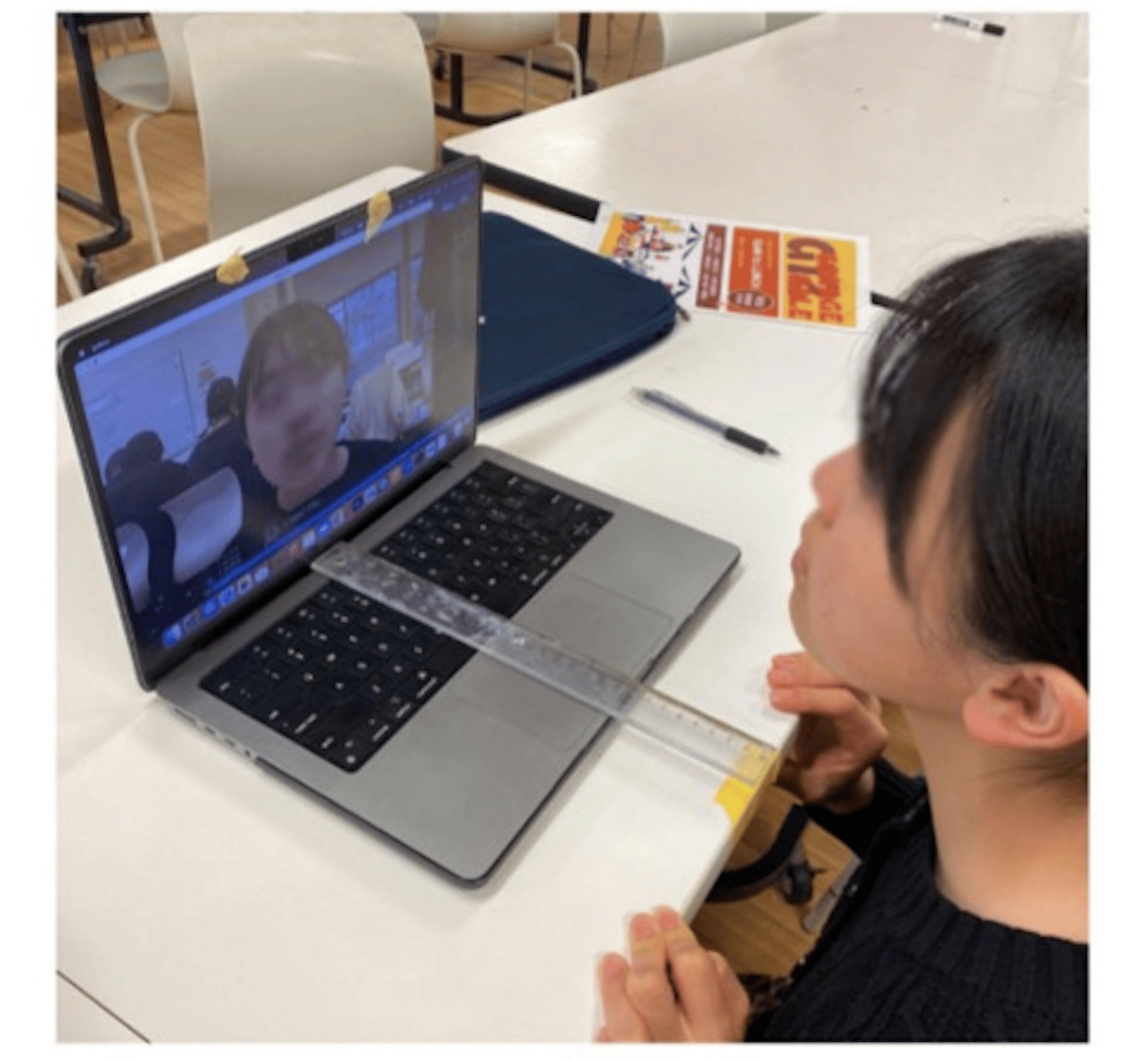
Procedure for acquiring ocular position images from a subject. Written informed consent was obtained for the publication of the participant’s images.

Using this setup, the following instructions were given to each subject, and ocular position images of the cases were acquired. For each ocular position image, blue and green landmarks were estimated for the iris, and red arrows and their lengths were estimated according to the gaze degree using this algorithm. Each subject was positioned 30 cm from the camera, as measured with a ruler. While visually confirming that the built-in camera and the case's face were parallel, the subjects were instructed to “look directly at the camera,” and videos of ocular position were recorded (Figure 2).

## Results

A total of 12 subjects were included in the study, comprising two case subjects and 10 control subjects without ophthalmologic abnormalities. Participants were aged 15 to 16 years and included both females (n=9, 75%) and males (n=3, 25%). Most participants were of East Asian ethnicity, with two identified as Southeast Asian. Demographic details of all subjects are presented in Table 1.

**Table 1:** Participant demographics (n=12).

Group	Subject ID	Gender	Age (Years)	Ethnicity
Case Subjects	1	Female	16	East Asian
	2	Female	16	East Asian
Control Subjects	1	Female	16	South East Asian
	2	Male	15	East Asian
	3	Male	16	East Asian
	4	Female	16	East Asian
	5	Female	16	East Asian
	6	Female	16	East Asian
	7	Female	16	South East Asian
	8	Female	16	East Asian
	9	Male	16	East Asian
	10	Female	15	East Asian

Case 1

**Figure 3:**
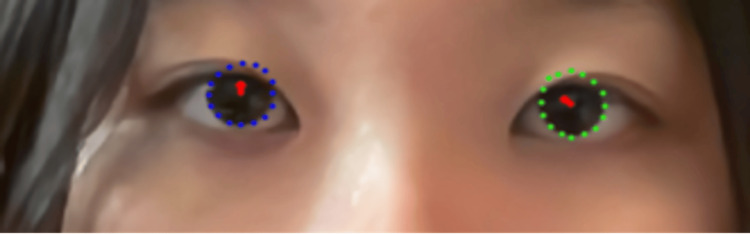
Case 1: no prior history of strabismus. The blue dotted line delineates the corneal margin of the right eye, the green dotted line delineates the corneal margin of the left eye, and the red arrow indicates the deviation from the corneal center. Written informed consent was obtained for the publication of the participant’s images.

A 16-year-old Japanese female presented with no significant medical history. She had a history of ophthalmologic examinations for school examinations and contact lens prescriptions, but no strabismus or other ophthalmologic examinations were noted. In this algorithm, landmarks in the right and left eyes were recognized without issue, and the results were as follows: 4.3 degrees of gaze deviation in the right eye, and -0.5 degrees of gaze deviation in the left eye (Figure 3).

Case 2

**Figure 4:**
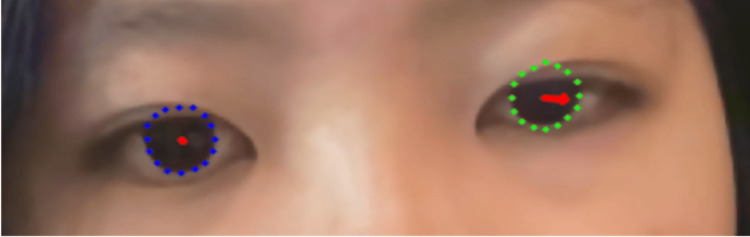
Case 2: exotropia in the left eye. The blue dotted line delineates the corneal margin of the right eye, the green dotted line delineates the corneal margin of the left eye, and the red arrow indicates the deviation from the corneal center. Written informed consent was obtained for the publication of the participant’s images.

A 16-year-old Chinese female was noted to have strabismus during a routine school screening in elementary school and was subsequently diagnosed with left exotropia with a deviation angle of 10 degrees at another hospital. However, she had no history of amblyopia treatment (details unknown) and was unaware of any visual function abnormalities. Again, landmarks in both eyes were recognized without issue, and the results were as follows: 0.7 degrees of gaze deviation in the right eye and -10.1 degrees of gaze deviation in the left eye (Figure 4).

The algorithm successfully recognized both eyes and estimated gaze angles in all cases. It was further tested on 10 subjects with no previous history of strabismus. The correlation between right and left eye gaze angles was assessed during dextroversion and levoversion.

**Figure 5:**
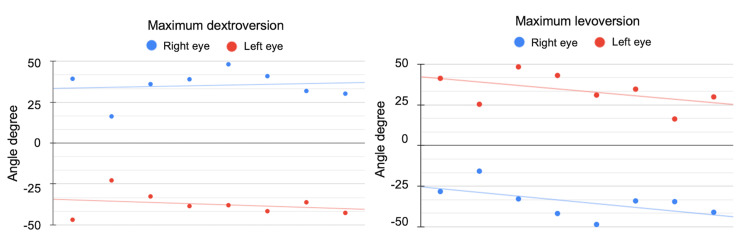
Correlation of left and right eye gaze angles during horizontal eye movements. Correlation of gaze angles between the left eye (red) and right eye (blue) during instructed rightward (dextroversion) and leftward (levoversion) gaze.

During dextroversion, the trend line indicated a strong correlation between the gaze angles of the right and left eyes (Spearman's r=0.961). The range of gaze angles in the primary position was ±0.5-5.7°, and the maximum eye movement in the secondary position (during version) was ±48.4°. Similarly, the trend line for levoversion also showed a very high correlation (Spearman's r=0.965). The range of gaze angles in the primary position was ±0.5-5.7°, and the maximum eye movement in the secondary position was ±48.4° (Figure 5).

## Discussion

We describe a novel application of DL-based gaze estimation for quantitative assessment of the strabismus angle.

First, “recognition of the right eye and left eye” and “estimation of the degree of eye gaze” were possible in all cases. This indicates that the algorithm is capable of “recognition of the right eye and left eye” and “estimation of the degree of eye gaze” using the “ocular position images” as input. When a case with no history of ophthalmologic diseases, including strabismus, was chosen as input, the detected value of gaze angle was low. On the other hand, when a case with strabismus was selected, the value of the gaze angle was high and similar to the previously diagnosed strabismus angle (Figure 3). This suggests that this algorithm has the potential for accurate gaze angle detection. In addition, although there were a few cases, a very high correlation was observed between the degree of gaze of the left and right eyes during rightward or leftward viewing, suggesting that this algorithm can be used for a variety of input images.

Most AI-based studies on the detection of strabismus and ocular position abnormalities have emerged from international research. de Figueiredo et al. reported the development of a diagnostic application for strabismus using ocular positional photographs from 110 cases [9], although their reported diagnostic accuracy ranged from 0.42 to 0.92, and validation of results was insufficient [9]. In contrast, Zheng et al. used 7,530 pediatric ocular positional photographs for training and developed a highly accurate photographic diagnostic algorithm (sensitivity 94.0%, specificity 99.3%, area under the receiver operating characteristic curve (AUC)=0.99) [10]. Using a large amount of training data, Zheng et al.’s algorithm boasts an accuracy that far exceeds the diagnostic capacities of a human ophthalmologist [10], though it was limited to detecting horizontal ocular positional abnormalities, such as exotropia or esotropia. Mao et al. further advanced accuracy using 5,797 images of corneal reflexes (sensitivity 99.1%, specificity 98.3%, AUC=0.998) [11].

Compared to these approaches, our algorithm has an advantage in terms of versatility regarding practical use. Since the input images are captured on video, it is considered useful for cases with torticollis or infants who have difficulty staying in a fixed position. Although further validation is required, it is also believed that the video input format will provide a certain level of accuracy even in cases where the input image is rotated in the camera (i.e., rotation in the sagittal and transverse planes, when torticollis is considered to be rotation in the coronal plane).

However, this study has several limitations. Developing an AI algorithm with sufficient accuracy for practical use typically requires several thousand to tens of thousands of training data [12]. The gaze estimation model for the basis of this algorithm was developed using 100,000 open-source eye images generated with UnityEyes (Computer Vision Laboratory, ETH Zurich, Zurich, Switzerland) [8]. Because these images were not originally collected for ocular position detection, the generalizability of the algorithm may be limited [12]. Compensatory head tilt can partially mask superior oblique palsy, rendering assessment in the primary gaze position alone insufficient to reliably demonstrate the condition. Moreover, in the absence of comparative testing using a synoptophore, this method should be regarded as a screening tool rather than a definitive diagnostic approach. In summary, the main limitations of this study include a small sample size, limited diversity of the training data, the absence of a clinical benchmark, and the lack of evaluation of rotation robustness. Therefore, future studies should verify whether the algorithm maintains accuracy across various ocular position images, including rotated image inputs. In addition, there are ethical and legal obstacles to overcome before practical application. Therefore, clinical studies for comparison with existing quantitation methods and validation are essential [13].

## Conclusions

This report demonstrates the feasibility of using a DL-based gaze estimation algorithm to quantify ocular misalignment in strabismus. The model successfully identified both eyes and estimated gaze angles from standard video inputs, showing a high correlation between both eyes in healthy participants and detecting deviations consistent with known strabismus diagnoses.

Although further validation and development are necessary before clinical application, this approach offers a practical, non-invasive, and accessible approach for strabismus assessment. Future work will focus on expanding the dataset, improving accuracy, and comparing performance against conventional diagnostic tools.

## References

[1] Luo J (2017). Strabismus treatment update for pediatricians [Article in Japanese]. J Ambul Gen Pediatr.

[2] Smith EL 3rd, Hung LF, Arumugam B, Wensveen JM, Chino YM, Harwerth RS (2017). Observations on the relationship between anisometropia, amblyopia and strabismus. Vision Res.

[3] Kfir J, Wygnanski-Jaffe T, Farzavandi S (2023). The impact of the first peak of the COVID-19 pandemic on childhood myopia control practice patterns among ophthalmologists-an international pediatric ophthalmology and strabismus council global perspective. Graefes Arch Clin Exp Ophthalmol.

[4] Lee HS, Park SW, Heo H (2016). Acute acquired comitant esotropia related to excessive Smartphone use. BMC Ophthalmol.

[5] Taylor K, Elliott S (2014). Interventions for strabismic amblyopia. Cochrane Database Syst Rev.

[6] Shimizu E, Tanaka K, Nishimura H (2024). The use of artificial intelligence for estimating anterior chamber depth from slit-lamp images developed using anterior-segment optical coherence tomography. Bioengineering.

[7] Newell A, Yang K, Deng J (2016). Stacked hourglass networks for human pose estimation. Lecture Notes in Computer Science.

[8] (2025). Gaze estimation with deep learning. https://github.com/david-wb/gaze-estimation.

[9] de Figueiredo LA, Dias JV, Polati M, Carricondo PC, Debert I (2021). Strabismus and artificial intelligence app: optimizing diagnostic and accuracy. Transl Vis Sci Technol.

[10] Zheng C, Yao Q, Lu J (2021). Detection of referable horizontal strabismus in children’s primary gaze photographs using deep learning. Transl Vis Sci Technol.

[11] Mao K, Yang Y, Guo C (2021). An artificial intelligence platform for the diagnosis and surgical planning of strabismus using corneal light-reflection photos. Ann Transl Med.

[12] Shimizu E, Kitazawa K, Murakami Y, Nakagawa T (2022). Basic research column 61) research and development of AI for anterior eye diagnosis. New Ophthalmol.

[13] Hasebe S, Ohtsuki H, Tadokoro Y, Okano M, Furuse T (1995). The reliability of a video-enhanced Hirschberg test under clinical conditions. Invest Ophthalmol Vis Sci.

